# An Alternative to Traditional Bedside Teaching During COVID-19: High-Fidelity Simulation-Based Study

**DOI:** 10.2196/33565

**Published:** 2022-05-09

**Authors:** Shereen Ajab, Emma Pearson, Steven Dumont, Alicia Mitchell, Jack Kastelik, Packianathaswamy Balaji, David Hepburn

**Affiliations:** 1 Hull York Medical School Hull University Teaching Hospitals NHS Trust Hull United Kingdom

**Keywords:** simulation, high fidelity, low fidelity, COVID-19, bedside teaching, undergraduate medical education, fidelity, medical education, medical student, review, innovation, risk, design, implementation

## Abstract

**Background:**

Bedside teaching is integral to medical education and has been highlighted to improve clinical and communication skills, as well as clinical reasoning. Despite the significant advantages of bedside teaching, its usage within medical education has been declining, and COVID-19 has added additional challenges. The pandemic has resulted in a significant reduction in opportunities to deliver bedside teaching due to risk of viral exposure, patients declining student interactions, and ward closures. Educators have therefore been required to be innovative in their teaching methods, leading to the use of online learning, social media platforms, and simulation. Simulation-based education allows for learning in a low-risk environment and affords the opportunity for deliberated repeated practice with case standardization. The results demonstrate that simulation-based training can increase students’ confidence, increase the rates of correct clinical diagnoses, and improve retention of skills and knowledge when compared with traditional teaching methods.

**Objective:**

To mitigate the impact of COVID-19 upon bedside teaching for third year students at Hull York Medical School amid closure of the cardiorespiratory wards, a high-fidelity simulation-based model of traditional bedside teaching was designed and implemented. The objectives of the teaching session were to enable students to perform history taking and a focused cardiorespiratory clinical examination in a COVID-19–safe environment using SimMan 3G.

**Methods:**

Four clinical teaching fellows with experience of simulation-based medical education scripted histories for 2 common cardiorespiratory cases, which were asthma and aortic stenosis. The simulation sessions were designed for students to take a focused cardiorespiratory history and clinical examination using SimMan 3G. All cases involved dynamic vital signs, and the simulator allowed for auscultation of an ejection systolic murmur and wheezing in accordance with the cases chosen. Key aspects of the pathologies, including epidemiology, differential diagnoses, investigations, and management, were summarized using an interactive PowerPoint presentation, followed by a debriefing session.

**Results:**

In total, 12 third year medical students undertook the sessions, and overall feedback was highly positive. Of the 10 students who completed the feedback questionnaires, 90% (n=9) felt more confident in their clinical examination skills following the teaching; 100% (n=10) of the students responded that they would recommend the session to a colleague; and implementation of regular simulation was frequently requested on feedback. These results are in keeping with the current literature.

**Conclusions:**

Bedside teaching continues to face ongoing challenges from the COVID-19 pandemic as well as declining patient recruitment and fluctuations in clinical findings. The support for simulation-based medical education is derived from high-quality studies; however, studies describing the use of this technology for bedside teaching in the undergraduate curriculum are limited. The authors describe a highly effective teaching session amid the pandemic, which allowed for maintenance of staff and student safety alongside continued education during a challenging time for educators globally.

## Introduction

The COVID-19 pandemic has posed significant challenges to undergraduate medical education. Teaching methods with patient-facing encounters such as bedside teaching have raised numerous difficulties with regards to exposure and testing of students, staff and patients, limited access to personal protective equipment, and strict social distancing requirements. The hurdles associated with conducting clinical placements amid the pandemic have been acknowledged by the UK Medical Schools Council [1]. In an attempt to ensure ongoing education during this time, web-based learning platforms have been increasingly adopted; however, not all areas of the undergraduate medical curriculum are best suited to this form of learning [2].

Bedside teaching has commonly been used to teach valuable core skills such as history taking and clinical examination, fostering effective communication skills and enabling professional relationships with patients and other health care professionals to be established [3]. Solutions to the difficulties associated with bedside teaching in the pandemic have been sought through the use of available technologies, including simulators. Simulation has been defined as a person, device, or set of conditions that attempts to present education and evaluation authentically [4]. Simulation-based teaching in health professions education has seen tremendous growth over the past 20 years, driven by factors including patient safety and the requirement for standardization of both training and assessment [5]. Simulation-based education, through various high-quality randomized controlled trials, has been shown to accelerate knowledge and skill acquisition including nontechnical skills, engage learners in deliberate practice, and provide a controlled low-risk learning environment [6-8].

Simulators can be classified according to the degree of realism known as fidelity. High-fidelity simulators provide the user with immersive and often complex scenarios with a high degree of realism [9]. Low-fidelity simulators replicate the real world to a lesser extent and include part-task trainers, for example, an inanimate model of a limb to allow learners to practice venipuncture skills [9]. In addition, medium- or intermediate-fidelity simulators provide greater authenticity than low-fidelity simulators but may require instructors to produce physiological signals displayed on monitors and therefore lack authenticity compared with high-fidelity simulators [10]. High- and low-fidelity simulators have been shown to have various advantages with recognized limitations. Weller has demonstrated that students have also derived benefit from medium-fidelity simulators, with feedback suggesting students found it useful to apply their knowledge in a safe environment using a structured approach, as well as developing their team-working skills [11]. High fidelity simulation can provide students with exposure to relevant clinical signs without requiring patient contact, while maintaining a high degree of realism [1]. Studies have demonstrated that students reported higher satisfaction and self- rated confidence scores when using high-fidelity simulators compared with low-fidelity models [12]. The advantages of simulation-based education have resulted in its incorporation into some of the medical school curriculums. Hull York Medical School has adopted simulation-based education for final year medical students and physician associate students using the high-fidelity simulator SimMan 3G.

SimMan 3G is an adult-size full body mannikin, which can be preprogrammed with adjustable parameters representing vital functions that can be visualized on a display monitor. Vital sign monitoring through the application of a saturation probe, electrocardiogram leads, and a blood pressure cuff onto the mannikin will allow for the parameters to be displayed on a monitor connected to the mannikin. The monitor displays dynamic heart rate, 3- and 12-lead electrocardiograms, blood pressure, mean arterial pressure, oxygen saturations, respiratory rate, and end tidal carbon dioxide values, allowing students immediate feedback when interventions are performed. The simulator allows for speech using a microphone and speaker, which is connected wirelessly to a separate control room where an instructor can simulate the patient’s voice. The technology audibly simulates cardiac murmurs, pathological respiratory sounds, and chest wall motion abnormalities as well as peripheral and central pulse palpation. Visually, the mannikin can simulate cyanosis, pupillary changes, diaphoresis, tongue oedema, pharyngeal swelling, trismus, and seizures.

Hull York Medical School delivers weekly bedside teaching for third year undergraduate students, facilitated by a team of clinical teaching fellows. During the academic year, students rotate through the 4 blocks of cardiorespiratory, gastroenterology, metabolic, and mental health. The focus of these teaching sessions is enabling students to develop their history taking and clinical examination skills in clinic environments. During the pandemic, several wards including the cardiology, cardiothoracic surgery, and respiratory medicine wards were closed to students due to outbreaks and to minimize risk of viral spread. The greatest impact was subsequently for those students undertaking their cardiorespiratory module. COVID-19 has therefore demanded educators to be innovative in their teaching methods, and the authors describe their personal experience of implementing novel simulation-based “bedside” teaching sessions to address the forementioned issues. It is likely that in the postpandemic era, technology will continue to play an important role in education [13]. The aim of this paper is to describe our experience of designing and delivering high-fidelity simulation-based teaching for history taking and clinical examination for simulated cases of asthma and aortic stenosis.

## Methods

### Materials

The team of 4 clinical teaching fellows, with experience in delivering simulation-based medical education at Hull York Medical school, designed and implemented a novel approach to bedside teaching. The aim of the teaching sessions was to provide continued high-quality education during the COVID-19 pandemic to third year medical students. The group of students that had already undertaken their cardiorespiratory block were identified, as this rotation received significant impact due to the pandemic, resulting in limited student exposure to patients from this specialty.

Two cases were chosen for the teaching sessions—asthma and aortic stenosis—due to their relative epidemiological prevalence and the ability to replicate clinical signs in the simulation suite (wheeze and ejection systolic murmur, respectively). Undergraduate medical students in their third year of education at Hull York Medical School were recruited via email by a student coordinator and offered electronic sign-up dates and times. The teaching sessions were delivered to pairs of students to allow sufficient time for each student to take a history and perform a focused respiratory or cardiac clinical examination. In addition, the small group sizes allowed for each student to observe their peers while maintaining compliance with social distancing requirements.

The format of the teaching sessions involved 2 students entering a simulation suite, accompanied by a teaching fellow to guide and support the session. In the suite, each student had the opportunity to take a history and perform a focused cardiorespiratory examination using the high-fidelity simulator SimMan 3G. The patient history was provided by a clinical teaching fellow in the control room in real time via a microphone linked to speakers in the simulation suite. The histories had been prescripted in order to allow for standardization of the cases and for learning objectives to be met. One teaching fellow was responsible for programming the simulator’s vital signs, providing the students with dynamic heart rate, blood pressure, oxygen saturations, respiratory rate, and temperature measurements. For each case the relevant positive clinical findings were simulated allowing for wheezing and an ejection systolic murmur to be auscultated. The limitations of the simulator were identified and therefore, to provide greater realism to the cases, visual aids were used in the form of printed photographs to demonstrate clubbing and a thoracotomy scar as well as props including a salbutamol inhaler. Each simulated session lasted approximately 45 minutes, and on completion of both cases, the students exited the suite and entered the debriefing room.

In the debrief room, a teaching fellow led a discussion of both cases with an emphasis on both individual and peer reflection and provided an opportunity for the students to ask questions. To summarize the key aspects of the topics covered during the simulation session, an interactive presentation was then delivered. The students spent approximately 45 minutes in the debrief room, which afforded the opportunity for the next pair of students to enter the simulation suite simultaneously. The equipment in the simulation room was cleaned between each pair of students, and all students performing the simulation wore appropriate personal protective equipment. Following the session, all students were sent a web-based feedback form and asked to rate their session in usefulness and relevance using the 5-point Likert scale (1=very poor, 5=very good), with additional white space fields to provide comments for qualitative feedback. The web-based form included standardized questions that Hull York Medical School uses to collect qualitative feedback, including learners who undertake regular simulation-based teaching in the fifth year of the medical program, as shown in Multimedia Appendix 1.

### Ethics Approval

This study was part of a quality improvement pilot project, and therefore no formal ethics board approval was required.

## Results

In total, 12 students completed the simulation-based teaching sessions, with 10 completing the feedback questionnaires. Analysis of the feedback demonstrated a very positive experience with an overall student self-rating score of 4.5 on a 5-point rating scale (1=very poor, 5=very good). The majority of students (n=9, 90%) felt more confident after the simulated bedside teaching, predominately with regards to clinical examination skills. Feedback also demonstrated that students found the combination of simulated scenarios alongside interactive presentations useful for their learning, and 100% (n=10) of students were keen to recommend the session to a colleague.

Student comments from qualitative feedback included “great opportunity for hands-on learning for practical skills” and “allows directed bedside teaching that is otherwise not available or not as easy to do with a real patient.” Moreover, 40% (n=4) of the students requested further simulation-based teaching sessions to be conducted using different clinical scenarios, and 1 (10%) student suggested “maybe we can have session like this in each block to practice for our end of block Objective Structured Long Examination Record.”

## Discussion

Our experience of delivering high-fidelity simulation-based teaching for third year undergraduate medical students demonstrated that the sessions were well received by students with high levels of learner satisfaction. In addition, the majority of students’ self-rated scores of confidence following the simulation sessions were high. Padilha et al [14] describe the development of knowledge as influenced by both student’s intrinsic factors as well as extrinsic factors such as satisfaction. Our experience is in keeping with studies described in the literature including that by Meyers et al [15], an observational pilot study that showed supplemental simulation-based training using a high-fidelity manikin improved overall satisfaction in preclinical medical students.

Physical examination is a vital skill for clinicians and is an essential component of high-quality patient care [16]. Traditionally, this skill has been taught in the clinical environment through bedside teaching; however, there are several challenges to this modality including declining patient recruitment, fluctuations in clinical findings during the course of treatment, and most recently the COVID-19 pandemic [16]. A recent systematic review by Dedeilia et al [12] analyzed these challenges imposed on medical and surgical education and summarized the innovations that have enabled the continuation of education in the era of COVID-19. Innovations include teleconferences and webinars, online learning, social media platforms, virtual consultations, virtual reality, and simulation [12].

Simulators vary with regards to fidelity. High-fidelity simulation, as described in our teaching, has several advantages including the ability to control physiological parameters, standardization of cases, and the relative ease of accessibility, and it allows students to contact with rare or life-threatening situations in a low-risk environment [17,18]. This can be contrasted with bedside teaching when patients who are available can be very variable and the “positive” examination findings can also be limited [17]. In addition, rarer and more complex patients may be too unwell to consult with students, or due to their inherent epidemiological rarity, may not be present within the hospital setting [19]. Simulation allows for the replication of these clinical profiles, providing students with hands-on exposure they may not otherwise gain [19]. Simulation has also been shown to reduce anxiety levels among medical students as shown by Yu et al [20]. The latter propose that students need to be repeatedly exposed to simulation for psychological stability and to develop competence [20]. These findings are further supported by Zheng et al [21], where structural integration of high-fidelity simulation in the cardiovascular physiology curriculum for undergraduate medical students proved successful with regards to student’s learning experiences and learning outcomes.

The most widely studied high-fidelity simulator is Harvey, a life-size manikin that simulates 27 cardiac conditions, which was introduced in 1968 by the University of Miami [18,22]. Giovanni et al [23] randomized 37 students to Harvey compared with CD tuition and assessed students via a 6-station objective structured clinical examination (OSCE), 6 weeks post teaching. The authors reported moderate (though not statistically significant) advantage in interpreting clinical signs in real patients in students trained with the simulator [23], as shown in Multimedia Appendix 2. There was no difference in diagnostic accuracy, which the authors postulated could be due to learning decay, resulting from the 6-week delay in testing. Giovanni et al [23] concluded that low-fidelity simulators also have a role and are associated with greatly reduced costs compared with high-fidelity simulators. However, studies by Anastakis et al [24] and Matsumo et al [17] have demonstrated that low-fidelity simulators had smaller gains than the high-fidelity simulator group (though not statistically significant).

O’Flynn [18] also notes that simulation training is able to increase student’s confidence but recognizes the risk of skill decay. In order to overcome this, Kneebone et al [19] propose distributed learning resources, allowing learners to access a range of simulation suitable for their level of training. Learners involved in simulation-based education, when compared with traditional learning, have shown that greater retention and simulation can provide valuable opportunities for interdisciplinary interactions [25]. Reed et al [26] demonstrated the long-term benefits of simulation-based training when 98% of students scored at or above the minimum passing standards on retesting 1 to 9 months after receiving teaching on core emergency medicine skills using the Laerdal SimMan.

Simulation-based medical education has been shown to have long-term beneficial outcomes by reducing inherent risk to patients and reducing the frequency of medical error, thereby improving patient care [19]. Bernardi et al [22] used the Kyoto-Kagaku patient simulator to train a group of fifth year medial students on cardiac auscultation. Simulation exposure significantly improved heart auscultation skills with mitral regurgitation being correctly identified by 87.9% of students versus 71.4% of non–simulation trained students (*P*=.02) [22]. Increased diagnostic accuracy following simulation training was also demonstrated by Perlini et al [16] after incorporating a 10-hour teaching session using the Harvey simulator for medical students and residents. They found that after simulation-based teaching, learners had a greater ability to recognize the correct cardiac diagnoses (from 11% to 72% *P*<.001) compared with baseline [16].

Gauthier et al [11] randomized 32 first year medical students to teaching modules with standardized patients or Harvey simulators. The authors found no difference in mean OSCE scores but a higher frequency of correct diagnoses among the students trained with standardized patients [11]. However, student feedback revealed Harvey offered superior clinical findings, and the authors concluded a combined teaching program would be ideal for transferability to patients [11]. Butter et al [27] have demonstrated transfer of skills and knowledge learnt through simulation training to the clinical environment. Learners having undergone simulation training accurately assessed 93.8% of simulated heart sounds (*P*<.001) compared with 73.9% accuracy among untrained students [28]. The authors subsequently advocate for simulation-based mastery learning programs, contending that they are a practice and feasible modality, allowing sufficient time for practice and compliant with competency-based accreditation requirements [28].

Studies using high-fidelity simulators other than Harvey have also shown favorable results. In 2019, Arangalage et al [29] delivered 28 hours per year of simulation-based teaching to over 400 students by incorporating a simulation-based course into the undergraduate medical curriculum. The authors used the Lifeform Auscultation Trainer and Smartscope to teach cardiac auscultation, blood pressure measurements, peripheral arterial examination, and the clinical examination of heart failure [29]. In keeping with the results of our study, Arangalage et al [29] reported that the majority of students provided positive feedback and found the teaching useful. They concluded that the simulation-based teaching facilitated educator-student and student-student interactivity with fulfilment of pedagogical objectives [29].

Simulation has also been used effectively to teach specialist skills to medical students. Scholz et al [30] compared a high-fidelity simulator to a wood-and-leather phantom to teach intrapartum care to 46 undergraduate students. Students using the high-fidelity simulator felt better prepared for obstetric house jobs and performed better in obstetric skills evaluations [30]. Siassakos et al [25] conducted an exploratory randomized controlled trial involving 24 fourth year medical students randomizing 1:1 to hybrid simulation training or small-group tutorials to teach management of shoulder dystocia. The authors’ results demonstrated that students having undergone hybrid simulation training had significantly higher median total patient perception scores (11 vs 9, respectively; *P*=.02) and significantly higher median communication scores (4 vs 3; *P*=.01) compared with those who underwent small-group tutorials [25]. The time dedicated to debriefing and the provision of immediate feedback are also considered a significant strength of simulation-based teaching, an opportunity that is often lacking in the clinical setting [9]. According to Riaz et al [31], over 90% of students found debriefing a useful component.

A significant barrier to using high-fidelity simulation is the high costs involved. Karnath et al [32] overcame this by using transportable simulators (blood pressure simulator and palpable pulse simulator) to effectively deliver a cardiopulmonary module for second year medical students [32]. Students were assessed through an OSCE; 80% of the students accurately measured the blood pressure, and cardiopulmonary auscultation proficiency showed average recognition of 60% for cardiac abnormalities and 88% for pulmonary sounds [32].

The limitations of simulation-based teaching are recognized. This method of teaching is resource intensive, both with regards to staff and the inherent high costs involved with high-fidelity simulators [33]. Simulation-based teaching has a higher staff and technological requirement, in addition to requiring technical knowledge to run the simulation effectively [33]. These factors may explain why simulation-based education has not been as widely adopted in undergraduate education compared with postgraduate training. Hull York Medical School has already incorporated simulated-based teaching into the undergraduate program for final year students. The authors propose that the use of simulation for other year groups may also be advantageous as demonstrated by the self-rated responses from third year students and allows for more efficient use of this expensive resource.

Limitations of the simulation technology also require consideration. In clinical practice, patients may present atypically, and a disadvantage of simulation-based bedside teaching is that these subtle nuisances and atypical presentations are not conveyed as well as when compared with a true patient presentation [15]. Simulation-based teaching may therefore not always represent a suitable alternative, particularly when there is advocation for preserving bedside teaching even in the face of new technologically assisted learning methods, with beliefs being held that nothing can simulate real patient encounters [20].

The support for simulation-based medical education is derived from high-quality studies (Multimedia Appendix 2); however, studies describing the use of this technology for bedside teaching in the undergraduate curriculum are limited [9,18,22,28,31,33-37]. We have described a successful teaching session, well received and enjoyed by the students with increased self-rated confidence scores in keeping with other studies [22,29,33]. During the unprecedented times of the pandemic, alternatives to bedside teaching were grossly limited. The authors describe a highly effective teaching session amid the pandemic, which allowed for maintenance of staff and student safety alongside continued education during a challenging time for educators globally. The teaching sessions allowed for learning in a safe controlled learning environment while meeting learning objectives. The cases chosen represent common pathologies, and with careful design and planning, future scenarios could incorporate more complex and rarer patients to allow for a more diverse learning experience. Simulation-based education is a useful adjunct to traditional teaching modalities providing an immersive and highly interactive learning environment that more accurately reflects the clinical experience [9]. The use of emergent technology is most likely to be an indispensable component of post–COVID-19 undergraduate medical education [21].

## Appendix

Multimedia Appendix 1 Year 3 simulation-based bedside teaching session feedback questionnaire.

Multimedia Appendix 2 Studies using simulation in undergraduate medical education.

## Abbreviations

OSCE: objective structured clinical examination
