# Supplementary material for: An Alternative to Traditional Bedside Teaching During COVID-19: High-Fidelity Simulation-Based Study
Source: JMIR Med Educ. 2022 May 9;8(2):e33565. doi: 10.2196/33565 (PMC9089324; doi:10.2196/33565)
Supplement: Multimedia Appendix 1 [file mededu_v8i2e33565_app1.docx]

**Multimedia Appendix 1**. Year 3 simulation-based bedside teaching session feedback questionnaire.

We would really appreciate your feedback following today's Year 3 Cardiorespiratory Bedside Simulation session, in order for us to know what you found useful and also if there's anything we could improve on for next time. Thank you for your feedback.

**On a scale of 1 (very poor) to 5 (very good), how did you find the session overall?**

1 (very poor)

2 (poor)

3 (neutral)

4 (good)

5 (very good)

**On a scale of 1 (not at all useful) to 5 (very useful), how useful did you find the session?**

1 (not at all useful)

2 (not very useful)

3 (neutral)

4 (somewhat useful)

5 (very useful)

**On a scale of 1 (not at all relevant) to 5 (very relevant), how relevant did you find the content of the session?**

1 (not at all relevant)

2 (not very relevant)

3 (neutral)

4 (somewhat relevant)

5 (very relevant)

**On a scale of 1 (very poor) to 5 (very good), how relevant did you find the quality of teaching received overall?**

1 (very poor)

2 (poor)

3 (neutral)

4 (good)

5 (very good)

**Do you feel more confident on the topics covered following the session?**

Yes

No

Unsure

**Would you recommend this session to a friend?**

Yes

No

**What were the good points?**

**What could be improved for future sessions?**

**Any additional comments?**
