# Supplementary material for: An Alternative to Traditional Bedside Teaching During COVID-19: High-Fidelity Simulation-Based Study
Source: JMIR Med Educ. 2022 May 9;8(2):e33565. doi: 10.2196/33565 (PMC9089324; doi:10.2196/33565)
Supplement: Multimedia Appendix 2 [file mededu_v8i2e33565_app2.docx]

**Multimedia Appendix 2.** Studies using simulation in undergraduate medical education.

| Author | Learners + Intervention | Drive for study | Simulator type | Outcomes Measured | Findings |
| --- | --- | --- | --- | --- | --- |
|  |  |  |  |  |  |
| Gauthier et al  [11] | 32 1^st^ year medical students  Focused cardiac clinical examination | Can Harvey replace standardised patients in teaching physical examination skills? | Standardised patients vs Harvey | OSCE scores and student feedback | No difference in mean OSCE score.  Standardised patient groups had higher frequency of correct diagnosis. |
| Perlini et al  [16] | 657 students: 3^rd^ and 4^th^ year medical students and residents  Cardiac auscultation | Low availability of “typical” cardiac patients for bedside teaching | Harvey | Ability to assess five different cardiac diagnoses | Statistically significant improvement in recognition of correct cardiac diagnoses after simulation training |
| Bernardi et al  [22] | 104 5^th^ year medical students  Cardiopulmonary auscultation and heart diagnoses | Does short individual simulator training could improve cardiopulmonary auscultation skills? | Kyoto-Kagaku | Ability to correctly identify 3 cardiac and 5 lung sounds on the simulator | Simulation significantly improved cardiac auscultation skills with mitral regurgitation correctly identified by 89.7% of simulation trained students vs 71.4% of non simulation trained students (P=0.02) |
| Giovanni et al  [23] | 37 3^rd^ year medical students (+10 students in pilot study)  Identifying heart sounds | Lack of studies contrasting low and high fidelity simulators | Harvey versus low fidelity simulator (CD) | Observed stations scored on communication skills and examination skills using 5 point scale | Slightly better identification of heart sounds with high-fidelity simulation group, (not statistically significant) |
| Siassakos et al  [25] | 24 4^th^ year medical students  Shoulder dystocia management | To assess whether simulation can improve medical students’ skills | Limbs and Things mannequin | Communication skills via a validated scale + questionnaires | Students having simulation training had significantly higher median total patient perception scores compared with small group tutorials (11 vs 9 respectively P=0.0239) |
| Reed et al  [26] | 135 4^th^ year medical students  Core skills in emergency medicine | Simulation based mastery to improve procedural skills | Laerdal SimMan | Multiple choice computer-based skill-related quiz | Statistically significant improvement in core clinical skills after simulation training (P<0.001).  Retention testing: 98% of students scored at or above minimum passing score on retesting 1-9 months later |
| Butter et al  [27] | 108 3^rd^ and 4^th^ year medical students  cardiac auscultation | Trainee deficiency in cardiac auscultation skills | Harvey + computer tutorial vs control | Diagnostic accuracy with real patient and simulated heart sounds | Significantly higher cardiac auscultation accuracy with simulation training compared with untrained students (93.8% vs 73.9% P<0.001) |
| Arangalage et al  [29] | 816 2^nd^ year medical students  Cardiovascular diagnostics course | Decline in bedside teaching opportunities | SAM Basic® with a SimScope® stethoscope, Cardionics, Texas, USA | Feedback from students and educators | Improved student appreciation (session 1: 77% vs. 98%, session 2: 89% vs. 98%; p < 0.0001) and improved educator satisfaction (session 1: 84% vs. 98%, p = 0.007; session 2: 82% vs. 98%, p = 0.01) |
| Scholz et al  [30] | 63 students  Intrapartum care | impact of a high-tech, high-cost environment on medical students ’ learning experience and acquisition of obstetric skills | Noelle S575 | Students self-assessment + 3 performance tests (obstetric skills, cardiotocography tracing (CTG), obstetric decision making) | Greater student satisfaction with high fidelity simulator vs low fidelity. Better clinical examination with high fidelity experience. Equivalent results in decision making with low and high fidelity simulation. |
| Riaz et al  [31] | 150 5^th^ year medical students  Clinical skills in obstretics and gynaecology and internal medicine | To determine student satisfaction with simulation | iStan and Lucina (high fidelity), park task trainers including OpthoSim | Student feedback | Majority found simulation relevant to clinical practice (100% vs. 92.9%; P <0.001) and debriefing session useful (98.1% vs 94.8%; P = 0.015) |
| Karnath et al  [32] | 184 2^nd^ year medical students  Cardiopulmonary diagnosis and auscultation | Decrease in bedside teaching | Harvey, blood pressure simulator, palpable pulse simulator, CD roms | Skills OSCE | 80% of students accurately measured blood pressure, 60% average proficiency for cardiac abnormalities and 88% for pulmonary sounds |
